# Telemedicine for Potential Application in Austere Military Environments: Neurosurgical Support for a Decompressive Craniectomy

**DOI:** 10.1093/milmed/usae094

**Published:** 2024-03-28

**Authors:** Pieter W Stark, O J F van Waes, John S Soria van Hoeve, Boudewijn L S Borger van der Burg, Rigo Hoencamp

**Affiliations:** Trauma Research Unit, Department of Surgery, Erasmus MC University Hospital, Rotterdam, South-Holland 3015 GD, the Netherlands; Department of Surgery, Alrijne Hospital, Leiderdorp, South-Holland 2353 GA, the Netherlands; Trauma Research Unit, Department of Surgery, Erasmus MC University Hospital, Rotterdam, South-Holland 3015 GD, the Netherlands; Defense Healthcare Organization, Ministry of Defense, Den Haag, South-Holland 2511 CB, the Netherlands; Department of Neurosurgery, Erasmus MC University Hospital, Rotterdam, South-Holland 3015 GD, the Netherlands; Department of Surgery, Alrijne Hospital, Leiderdorp, South-Holland 2353 GA, the Netherlands; Trauma Research Unit, Department of Surgery, Erasmus MC University Hospital, Rotterdam, South-Holland 3015 GD, the Netherlands; Department of Surgery, Alrijne Hospital, Leiderdorp, South-Holland 2353 GA, the Netherlands; Defense Healthcare Organization, Ministry of Defense, Den Haag, South-Holland 2511 CB, the Netherlands; Department of Surgery, Leiden University MC, Leiden, South-Holland 2333 ZA, the Netherlands

## Abstract

**Introduction:**

The main goal of this study was to assess the feasibility of a head-mounted display (HMD) providing telemedicine neurosurgical support during a decompressive craniectomy by a military surgeon who is isolated from readily available neurosurgical care. The secondary aim was to assess the usability perceived by the military surgeon and to evaluate technical aspects of the head-mounted display.

**Materials and Methods:**

After a standard concise lecture, 10 military surgeons performed a decompressive craniectomy on a AnubiFiX-embalmed post-mortem human head. Seven military surgeons used a HMD to receive telemedicine neurosurgical support. In the control group, three military surgeons performed a decompressive craniectomy without guidance. The performance of the decompressive craniectomy was evaluated qualitatively by the supervising neurosurgeon and quantified with the surgeons’ operative performance tool. The military surgeons rated the usability of the HMD with the telehealth usability questionnaire.

**Results:**

All military surgeons performed a decompressive craniectomy adequately directly after a standard concise lecture. The HMD was used to discuss potential errors and reconfirmed essential steps. The military surgeons were very satisfied with the HMD providing telemedicine neurosurgical support. Military surgeons in the control group were faster. The HMD showed no hard technical errors.

**Conclusions:**

It is feasible to provide telemedicine neurosurgical support with a HMD during a decompressive craniectomy performed by a non-neurosurgically trained military surgeon. All military surgeons showed competence in performing a decompressive craniectomy after receiving a standardized concise lecture. The use of a HMD clearly demonstrated the potential to improve the quality of these neurosurgical procedures performed by military surgeons.

## INTRODUCTION

Due to the nature of their work and the hostile environments they encounter, military personnel are exposed to an increased risk for traumatic injuries, especially traumatic brain injury (TBI). It is estimated that 20% of US service members engaged in Operation Iraqi Freedom and Operation Enduring Freedom may have sustained TBIs, varying from mild, moderate to severe.^1–3^

Increased mass within the fixed volume of the osseous cranium caused by hemorrhage or cerebral edema accompanies a high risk of developing elevated intracranial pressure. Following guidelines, the management of severe TBI (sTBI) involves a multi-step approach to regulate elevated intracranial pressure.^4^ This approach incorporates therapeutic interventions such as hyperosmolar therapy, avoidance of hypotension, normothermia maintenance, and hyperthermia prevention.^4^ If these steps are insufficient, cerebrospinal fluid diversion through an external ventricular drain, a relatively low-risk procedure, can temporize elevated intracranial pressure and should be considered prior to, or in conjunction with, a decompressive craniectomy (in short craniectomy).^5^ A craniectomy is a surgical procedure, in which, part of the skull is removed to effectively lower the elevated intracranial pressure and to remove any visible hematoma.^6,7^

In armed conflicts, damage control surgery is performed by forward surgical teams or by medical experts in support hospitals.^8^ Austere and high-risk environments demand that military surgeons possess a diverse skill set, often extending beyond their traditional scope of practice. Craniectomy has been established as the standard of care for high-energy wartime sTBI refractory to medical intervention, as supported by multiple publications drawing on the NATO and US Military experiences in Afghanistan and Iraq.^9–12^ However, very few formalized curricula are available to train a military surgeon in neurosurgical procedures, and exposure to the performance of a craniectomy is minimal, which may lead to a decreased likelihood of survival after sTBI if no neurosurgeon is available on-site.^8,13,14^ Options for evacuation are also sparse, which can cause a delay in receiving proper treatment. Diagnosing sTBI and identification of neurosurgical indications for craniectomy present additional challenges in austere military environments. Here, military surgeons must rely on clinical examination because civilian diagnostic modalities, including CT imaging, often are unavailable. As a consequence of these limitations, sTBI is often managed conservatively in armed conflicts, while performing a craniectomy early may enhance the likelihood of survival and favorable outcomes.^10,11,15^

Better neurosurgical training for the military surgeon could increase the preparedness for a craniectomy.^16,17^ Telemedicine neurosurgical support is recommended as an adjunct for military surgeons during craniectomies.^8,16,18^ A head-mounted display (HMD) providing telemedicine neurosurgical support might lead to a more adequate performance of a craniectomy performed by military surgeons and reduce the occurrence of errors. A HMD is a wearable device that projects data onto lenses while capturing images and videos through an integrated front-facing camera.^19^ It supports telestration, a technique defined as the real-time annotation of images or videos enabling the consulting neurosurgeons to remotely observe and provide precise instructions during neurosurgical procedures.^20^ This innovative approach eliminates the need for physical supervision on-site and enables hands-free communication between the military surgeon and the supervising neurosurgeon during the craniectomy. The goal is to bridge the gap between urgent neurosurgical procedures and remote neurosurgical expertise.

The main aim of this study was to assess the feasibility of a HMD providing telemedicine neurosurgical support during a craniectomy performed by non-neurosurgically trained military surgeons. The secondary aim was to assess the perceived usability of the HMD by military surgeons and to evaluate the technical aspects of the HMD.

## METHODS

This feasibility study was conducted at the Skillslab & Simulation Center of Erasmus University Medical Center Rotterdam, the Netherlands. The AnubiFiX-embalmed post-mortem human specimens^21,22^ used for the interventions were donated for scientific research and medical training at the Anatomy department of Skillslab, Rotterdam. These specimens were part of a national body donation program approved in compliance with Dutch law and regulations. The study protocol received ethical approval from the Dutch Ministry of Defense and the Institutional Review Board of Alrijne Hospital, the Netherlands (NWMO 17-15, 17.409rt.tk).

A neurosurgeon with expertise in neurotrauma participated as supervisor. Participants were selected from a group of non-neurosurgically trained military surgeons who performed a craniectomy as part of updating their military surgical skills. All military surgeons provided informed consent for their participation in the study.

**FIGURE 1.**
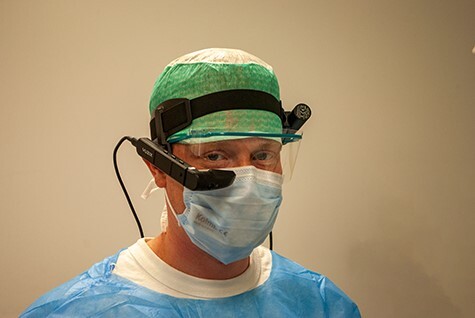
An example of a military surgeon wearing the Vuzix M400 HMD.

The Vuzix M400 (Vuzix Corporation, Rochester, NY, United States) was selected as HMD to conduct this study (Fig. 1). Two MacBook Air 2017 devices (Apple, Cupertino, CA, United States) were used to observe and telestrate the surgical procedures performed by the military surgeons. The HMDs and MacBooks were connected to a WPA-2-encrypted WiFi network. Gemvision software (Gemvision, Rotterdam, the Netherlands) was installed on both MacBooks and on the HMDs used. Gemvision software is certified with ISO 27001 and NEN 7510 standards for information security in health care.

### Study Procedures

Before conducting the craniectomies, all military surgeons attended a standardized concise lecture, which was part of a formalized curriculum based on microteaching principles. This lecture covered basic brain anatomy, indications for craniectomy, surgical techniques for craniectomy, and guidance on HMD use. After the lecture, all military surgeons received a demonstration of the essential steps of a craniectomy and hands-on instruction in the safe and effective use of a high-speed cranial drill. This drill featured a safety mechanism to halt drilling upon dura contact. All the steps and the surgical procedure performed by the neurosurgeon have been benchmarked as the gold standard in neurosurgery.

**FIGURE 2.**
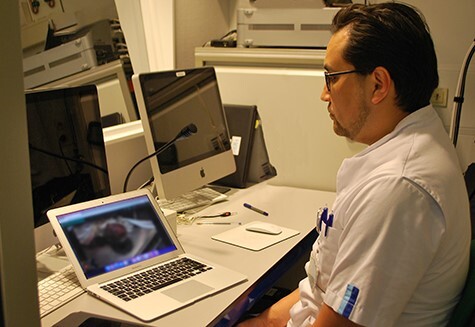
An example of the supervising neurosurgeon supervising the craniectomies.

The military surgeons were divided into two groups: one group performed a craniectomy while using a HMD (referred to as the “HMD group”), and the other group performed a craniectomy without guidance (referred to as the “control group”), according to a 2:1 ratio. The study was conducted in 45-minute time blocks. In the HMD group, two military surgeons performed a craniectomy per time block on a AnubiFiX-embalmed post-mortem human head. The supervising neurosurgeon observed, provided real-time input to the operative military surgeons, and assessed their performance remotely, without direct contact with the military surgeons (Fig. 2). In the control group, the same supervising neurosurgeon observed the procedure in the operating room to evaluate tissue handling, instrument use, and safety but did not provide direct instructions. Once the craniectomy was completed, the supervising neurosurgeon examined the AnubiFiX-embalmed post-mortem human head in the operating room to assess adequacy and discuss findings with the performing military surgeon, offering suggestions for improvement.

### Outcome Measures and Statistical Analysis

The main study parameter was the adequacy of the craniectomy performed by the military surgeon. This was assessed qualitatively by the researchers and the supervising neurosurgeon. Quantitative assessment was conducted by the supervising neurosurgeon, using a modified version of the surgeons’ operative performance tool (SOPT), a 9-item validated tool to measure surgical performance.^23^ Items were scored on a scale from 1 to 5, with 1 indicating poor performance, 3 indicating competence, and 5 indicating high performance.

The SOPT, usually applied to real-world cases with actual pathology, was modified for this study with post-mortem human specimen lacking cranial pathology. Therefore, the items “difficulty of the case” and “use of assistants” were excluded from final analysis because “difficulty of the case” might not accurately reflect the complexity of scenarios without cranial pathology, and “use of assistants” could not be assessed in the control group. The item “preparing for the craniectomy procedure” included correct head positioning with the sagittal plane 15° horizontal to the floor and making a question-mark-shaped frontotemporoparietal skin incision, larger than the intended craniectomy area.^24,25^ “Respect for tissue” emphasized minimizing mechanical trauma during bone removal, gentle opening of the dura mater, and careful handling of the brain parenchyma.^24,25^ “Time and motion” focused on use of time and economical movement. Motion included removing a bone flap size of at least 12 × 15 cm, along with proper dural opening and accurate middle fossa decompression.^4,24^ “Flow of operation and forward planning” included effective management of cerebrospinal fluid.^24^ “Ability to adapt to anatomical variances” included adjustments to neurosurgical techniques based on varying bone density and the course of arteries and venous sinuses, and performing sinus exenteration when required.^24^ “Knowledge of materials and instruments” included the appropriate use of neurosurgical instruments and correct operation of the high-speed cranial drill. “Overall performance” assessed the overall performance of the military surgeons during craniectomy. The SOPT was completed by the supervising neurosurgeon after each craniectomy procedure. Median and range values for the applicable SOPT items were calculated, and performance scores were compared between groups using the Mann-Whitney U test. Due to non-normal distributions of performance scores, as determined through visual inspection, differences between the two groups were calculated using mean rank.

The secondary study parameters included an assessment of the HMD usability using the telehealth usability questionnaire (TUQ), a validated tool for evaluating telehealth services.^26^ It has 21 items with each item utilizing a 7-point Likert scale, ranging from 1 (least usable) to 7 (most usable). Military surgeons in the HMD group completed the TUQ after each craniectomy. Median and range values were determined for each TUQ question. The independent samples *t*-test was employed to analyze total procedure time between the HMD group and the control group. The data were found to be normally distributed according to the Shapiro-Wilk test (*P* > .05). Technical defects were documented and reviewed by the research team. Statistical analyses were conducted in collaboration with a statistical expert using the Statistical Package for the Social Sciences (V.28, 2021, IBM Corporation, Armonk, NY, United States).

## RESULTS

A total of 7 military surgeons were enrolled in the HMD group and 3 military surgeons were enrolled in the control group.

### Preparation and Procedure

**FIGURE 3.**
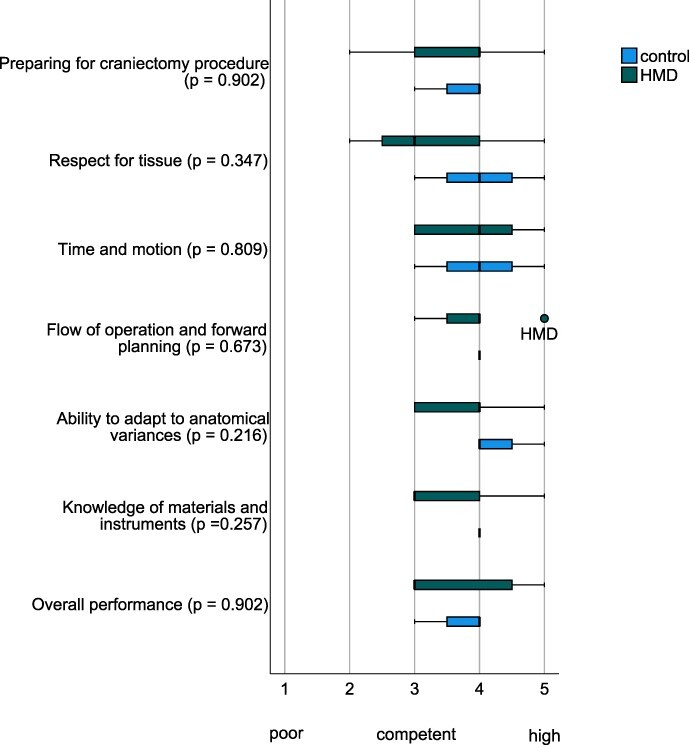
Surgeons’ Operative Performance Tool scores (median, range)*.

All military surgeons prepared for the craniectomy using their own judgment with only minimal adjustments by the supervising neurosurgeon [Fig. 3; HMD group: median 4 (range 3), control group: median 4 (range 1)]. Both groups carefully handled brain parenchyma and gently opened the dura mater, but military surgeons in the HMD group occasionally caused some inadvertent damage to the skull during bone flap removal [Fig. 3; HMD group: median 3 (range 3), control group: median 4 (range 2)]. Qualitative observations showed that the supervising neurosurgeon effectively utilized the HMD to give recommendations on the optimal head positioning and to determine the precise extent of the skin incision. The supervising neurosurgeon also observed that military surgeons used the HMD to receive recommendations on safely opening the dura mater without damaging the brain parenchyma.

* Mann-Whitney U test..0 HMD: outlier.

### Flow

Military surgeons in the HMD group and the control group demonstrated efficient time management and movement economy with proper dural opening and accurate middle fossa decompression [Fig. 3; HMD group: median 4 (range 2), control group: median 4 (range 2)]. Both groups planned the course of the craniectomy with steady progression of operative procedures [Fig. 3; HMD group: median 4 (range 2), control group: median 4 (range 0)]. Military surgeons in both groups were able to adapt the procedure to anatomical variances with minimal guidance and instruction [Fig. 3; HMD group: median 4 (range 2), control group: median 4 (range 1)]. Qualitative observations showed that the HMD was used to reconfirm essential steps during the craniectomy, discuss cerebrospinal fluid management techniques, and inform about sinus exenteration indications in real-world cases.

### Instrument Use and Overall Performance

Military surgeons in the HMD group were familiar with most neurosurgical instruments, while military surgeons in the control group had a slightly more comprehensive familiarity with the neurosurgical instruments [Fig. 3; HMD group: median 3 (range 2), control group: median 4 (range 0)]. Qualitative observations revealed that military surgeons utilized the HMD for two main purposes: to recall instructions related to the usage of the high-speed cranial drill and to improve ergonomic movements during the use of a Gigli saw. Military surgeons in the HMD group were rated as competent in their overall performance. In comparison, those in the control group were rated slightly higher, as indicated by the median scores [Fig. 3; HMD group: median 3 (range 2), control group: median 4 (range 1)]. There were no significant differences in SOPT outcomes between the HMD group and the control group (Fig. 3).

The mean procedure time in the HMD group was 29 minutes and 10 seconds (SD 11 minutes and 23 seconds), significantly longer than the control group, which completed procedures in a mean time of 12 minutes (SD 1 minute and 8 seconds) (*P* = .036).

**FIGURE 4.**
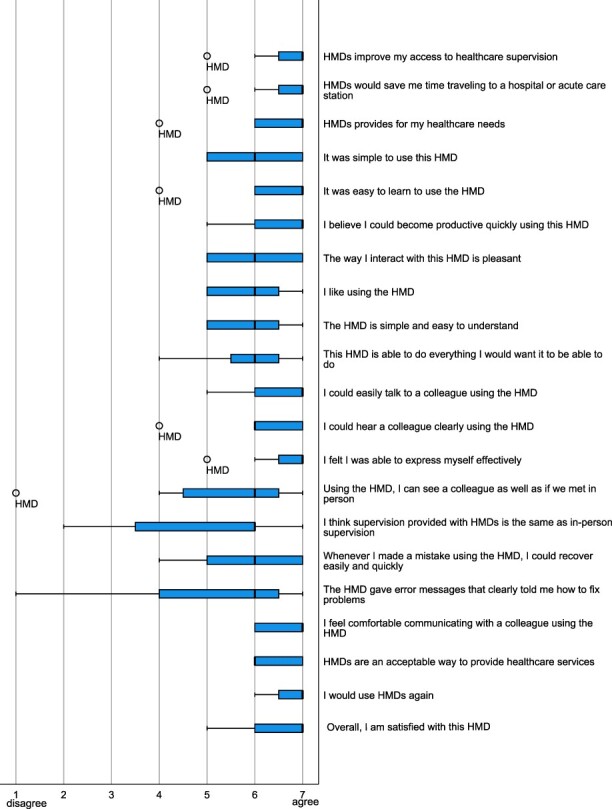
Telehealth Usability Questionnaire scores (median, range).

Overall, military surgeons expressed a high level of satisfaction with the HMD for receiving telemedicine neurosurgical support (Fig. 4: median 7, range 2). They strongly agreed that the HMD is a valuable tool for telemedicine neurosurgical support (Fig. 4: median 7, range 2). Learning to use the HMD was easy for military surgeons (Fig. 4: median 7, range 3) and it was straightforward to communicate with the neurosurgeon (Fig. 4: median 7, range 2). Additionally, all military surgeons strongly agreed that they would use the HMD again (Fig. 4: median 7, range 1).

0 HMD: outlier.

The quality of the connection was deemed satisfactory, with military surgeons reporting clear communication with the neurosurgeon throughout the procedure, and the HMD screen remained visible. No adverse effects related to HMD usage were reported. The supervising neurosurgeon maintained a clear overview of each military surgeon’s field of view, and anatomical details were distinguishable on the computer screen.

## DISCUSSION

This study evaluated the feasibility of using a HMD to provide telemedicine neurosurgical support for a military surgeon when performing a craniectomy. Following a standardized, concise lecture, all craniectomies were performed adequately. The HMD facilitated real-time discussion of potential errors and confirmation of essential steps during the procedure. The military surgeons reported high levels of satisfaction with the HMD for telemedicine neurosurgical support, underscoring its user-friendliness and practicality in supporting surgical performance. Overall, this study shows the feasibility of using a HMD providing telemedicine neurosurgical support for a non-neurosurgical trained military surgeon when performing a craniectomy and demonstrates their potential to enhance the quality of neurosurgical procedures.

Currently, the training and exposure to craniectomy procedures for all (military) surgeons is minimal.^8,13,14^ While participation in trauma masterclasses has shown to increase self-efficacy in trauma management competencies for trauma care providers, including military surgeons,^27^ the longevity of these competencies—specifically, their retention after 2 years—is uncertain.^28^ It is expected that HMDs providing telemedicine neurosurgical support will be particularly beneficial when recent training in neurosurgical procedures is lacking. In austere environments where no neurosurgeon is available on site, telemedicine neurosurgical support is recommended for military surgeons undertaking neurosurgical procedures.^8,16,18^ This feasibility study also shows that such support might enhance the safety and technical outcomes of craniectomies. However, these results do not provide insight into the long-term retention of these compentencies. Our research group has previously demonstrated the feasibility of HMDs for providing telemedicine support to combat medics performing a two-incision lower leg fasciotomy.^29^ To better understand competency retention, determine the optimal window for refresher training, and identify which subgroups of military surgeons would most benefit from HMD use, a follow-up study should be conducted after 2 years. While the diagnosis of sTBI and the identification of neurosurgical indications for a craniectomy were not within the scope of this study, the use of HMDs could potentially assist military surgeons in clinical examinations and in consulting with remote neurosurgeons regarding neurosurgical indications for a craniectomy.

Military surgeons in the control group completed the craniectomies faster. Those equipped with a HMD took longer, likely because they discussed potential errors and reconfirmed essential steps, such as verifying the accurate anatomical size for a craniectomy. Current guidelines do not specify an optimal duration for craniectomies.^4,30–32^ During the craniectomies, minor damage to the external surface of the skull was observed in some cases within the HMD group. It is important to clarify that this damage was superficial and, in real-world cases, would be expected to heal naturally without affecting the overall outcome of the procedure.

The HMD showed no hard technical errors. Future research should examine the added value of an HMD for providing telemedicine neurosurgical support to military surgeons in real austere environments, considering various connectivity options. Previous studies have shown the Vuzix M400’s effectiveness with a 4G wireless network in civilian emergencies.^33–35^ However, austere military environments, where a 4G wireless network is often unavailable, present different connectivity challenges. While the Vuzix M400 can be connected to satellite communications, such a connection has only been applied in test cases so far and has not yet been described in previous literature. In a future study, the positive outcomes observed in these test cases, specifically regarding bandwidth and latency performance in satellite communications, could be further evaluated in a real austere military environment.

This feasibility study had some limitations. While the small sample size of this study may limit the detection of significant differences, the primary objective of a feasibility study is not to establish statistical significance but rather to test the practicality and potential application of a new technology. The military surgeons reconfirmed several essential steps with the supervising neurosurgeon and responded to spoken feedback. However, functions such as telestration were not utilized. It was beyond the scope of this study to evaluate the impact of prior training on the full capabilities of the HMD. In this study, an AnubiFiX-embalmed post-mortem human head was used, which did not simulate acute bleeding or elevated intracranial pressure. Nevertheless, given the limited opportunities for military surgeons to practice craniectomies on actual patients, this model represents the best available approximation of a real case.

## CONCLUSION

It is feasible to provide telemedicine neurosurgical support with a HMD during a craniectomy performed by a non-neurosurgically trained military surgeon. All military surgeons showed competence in performing a craniectomy after receiving a standardized concise lecture. The use of a HMD clearly demonstrated the potential to improve the quality of these neurosurgical procedures performed by military surgeons.

## Data Availability

The data underlying this article will be shared on reasonable request to the corresponding author. All data are freely accessible.
